# Development and validation of a simple, sensitive enzyme immunoassay for quantification of androstenedione in bull plasma

**DOI:** 10.1186/s40781-014-0035-z

**Published:** 2015-04-05

**Authors:** Smrutirekha Mallick, BS Bharath Kumar, BS Prakash, Anjali Aggrawal, Sujata Pandita

**Affiliations:** Dairy Cattle Physiology Division, National Dairy Research Institute, Karnal, 132001 India; Division of Animal Sciences, Indian Council of Agriculture Research, Krishi Bhavan, 10001 New Delhi

**Keywords:** Androstenedione, Enzyme immunoassay, Bull, Plasma, Standardisation

## Abstract

As an alternative to radioimmunoassay a simple and highly sensitive enzyme immunoassay (EIA) was developed and validated for androstenedione quantification in plasma of Karan Fries bulls using second antibody coating technique. The wells of the microtitreplate were coated with affinity-purified goat immunoglobulin (antirabbit IgG) that binds the hormone specific antibody. The EIA was performed to analyze androstenedione directly in 40 μl of bull plasma. The androstenedione standards ranged from 0.20 to 200 pg/40 μl /well and the sensitivity of the assay was 5 pg/ml plasma. Serially diluted bull plasma containing high endogenous androstenedione showed good parallelism with bovine androstenedione standard curve. Intra- and inter-assay coefficients of variation (CV) were found to be 8 and 9%, respectively. Peripheral plasma androstenedione concentrations determined in young and adult bull samples ranged between 104–990 pg/ml and 184–2040 pg/ml, respectively.

## Background

Androstenedione is the common precursor of male and female sex hormones. Being the predominant steroid in case of young animals, measurement of androstenedione provides a useful marker of androgen biosynthesis. Several radioimmunoassay (RIA) procedures [1-4] for estimation of androstenedione were developed and standardised, however these procedures require an extraction step which entails the use of hazardous solvents. Additionally the use of a radioisotope limits the application to labs which possess license; other disadvantages include proper disposal of radioactive waste, use of expensive instrumentation and requirement of adequate lab space. Although a few enzyme immunoassay (EIA) procedures have been developed [5,6] in recent years, the direct coating of hormone specific antibody on microtitre plate employed by these procedures resulted in extensive usage of the expensive antiserum. Advantages of second antibody coating EIA technique over direct coating technique are reduced time dependent drift, less expensive, high precision and sensitivity. Hence a study was designed to develop a highly sensitive and specific EIA using second antibody coating technique for androstenedione determination in bovine plasma.

## Methods

### Enzyme immunoassay for androstenedione

The development of a heterologous competitive ELISA for androstenedione determination was done using 96-well flat bottomed polystyrene microtitre plates (Greiner Labotrechnik, Germany) using the second antibody coating technique and androstenedione–HRP as a label in unextracted bull plasma. The affinity-purified goat IgG (antirabbit IgG) was developed following the procedure of Anandlaxmi and Prakash [7]. The antiserum used in these assays was highly specific for androstenedione. Percentage cross-reaction of androstenedione antiserum with related steroids at 50% binding was determined.

### Preparation of hormone-free plasma and enzyme label

For preparation of androstenedione-free plasma, blood samples were collected from aged Karan Fries cows. Plasma was separated after centrifugation of blood and in-order to remove traces of androstenedione, it was treated with charcoal and dextran mixture as follows:

Mixture of activated charcoal (14 g) and dextran T-70 (1.4 g) per 100 ml plasma were taken and the mixture was washed with distilled water by thorough mixing, using a magnetic stirrer for overnight at room temperature. After allowing the mixture to stand for 5 min, the supernatant was discarded and the mixture was again subjected to the washing process for three more times at 8 h intervals to remove all traces of suspended fine charcoal particles. Cow plasma was then added to the washed charcoal–dextran mixture and mixed thoroughly for 2 h at 4°C. Then the mixture was centrifuged at 1500 × *g* for 1 h at 4°C and the supernatant was filtered to remove suspended particles. The filtrate was again centrifuged at 5000 × *g* for 1 h at 4°C and the supernatant was re-filtered. The plasma thus obtained was tested for androstenedione concentration in an assay. The concentration of androstenedione in this charcoal dextran stripped plasma was below detectable limit of the assay (<5 pg/ml). The androstenedione-free plasma was then stored at −20°C for future use.

Horseradish peroxidase (HRP; Serva, Germany) was used for coupling to androstenedione using the mixed anhydride method [8] with modifications [9].

### EIA procedure

Wells were coated with 0.63 μg of goat IgG antirabbit IgG dissolved in 100 μl of coating buffer (15 mM Na_2_CO_3_ and 35 mM NaHCO_3_, pH 9.6) per well of the microtitre plate. The plates were subsequently incubated overnight at 4°C. For blocking the remaining binding sites, 300 μl of 1% bovine serum albumin (BSA) in phosphate buffer was added to all the wells and incubated for 40–50 min at room temperature under constant shaking. The coated plates were then washed twice with 350 μl/well of washing solution (0.05% Tween 20) using an automated microtiter plate washer (Labsystem, Model: EL 50 8MS, USA). Then a two-dimensional titer determination for optimum dilution of each androstenedione–HRP conjugate and androstenedione antiserum was carried out. Antibody dilutions ranging from 1:1,000 to 1:640,000 and androstenedione–HRP dilutions of 1:5,000 to 1:60,000 were tested. The antibody titer of 1:8,000 and the androstenedione–HRP conjugate titer of 1:20,000 were found to be optimum, and OD_450_ of around 0.85 was achieved by using these titers. Then duplicates of 40 μl of unknown plasma sample or androstenedione standards prepared in charcoal–dextran treated hormone-free plasma ranging from 0.20 to 200 pg/40 μl/well were simultaneously pipetted into respective wells along with 100 μl of androstenedione–HRP conjugate diluted (1:20,000) in assay buffer with the aid of a dilutor dispenser. Then, 100 μl of androstenedione specific antiserum diluted in assay buffer (1:8,000) was added immediately to all wells except blank by a repeat dispenser.

The plates were kept in water bath for 1 h at 37°C and thereafter incubated overnight at 4°C. The plates were then washed four times with washing solution and incubated further in the dark for 40 min after addition of 150 μl of substrate solution per well (substrate buffer: 0.05 M citric acid, 0.11 M Na_2_HPO_4_ and 0.05% urea hydrogen peroxide: pH 4.0 adjusted with 5 N HCl; substrate solution: 17 ml substrate buffer plus 340 μl 3,3′,5,5′-tetramethyl benzidene; 12.5 mg/ml dimethyl sulfoxide; Sigma, USA). The reaction was stopped by the addition of 50 μl 4 N H_2_SO_4_ and the intensity of colour was measured at 450 nm with a 12-channel microtitre plate reader (Model: ECIL, Microscan, India).

### Parallelism between androstenedione standards and endogenous androstenedione in bull plasma

To determine the parallelism between androstenedione standards and endogenous androstenedione, plasma samples from two bulls containing high concentrations of endogenous androstenedione were serially diluted with EIA assay buffer to obtain plasma volumes of 40, 20, 10, 5, 1.25 and 0.63 μl. Parallelism was assessed between these serial dilutions of the bull plasma samples and androstenedione standards (ranging from 0.20 to 200 pg/40 μl/well, prepared in assay buffer) when run in an assay.

### Experimental animals and blood sampling

The experiment was conducted in the Artificial Breeding research Complex (ABRC); National Dairy Research Institute, Karnal, Haryana, India. Four adult Karan Fries bulls (3–3.5 yr) and Four Karan Fries bull calves (8–10 months) were selected and maintained in individual bull pens, under stall-fed condition and uniform managemental practices. Five blood samples were collected from all the animals in EDTA coated tubes by jugular vein puncture at 30 minutes interval for a period of 2 h. The samples (5 ml) were immediately cooled in ice and centrifuged at 4°C. The plasma obtained was stored at −20°C until hormone analysis by the procedure as described below. Animal experimentation methods were approved by the Institutional Animal Ethical Committee of National Dairy Research Institute (1705/GO/ac/13/CPCSEA Dt. 3/7/2013).

Recovery of the added androstenedione was determined by measuring androstenedione previously added to hormone free plasma. Results obtained by the developed EIA technique (un-extracted plasma) were compared with the RIA (commercially available kit) method in ether extracted plasma samples.

### Statistical analysis

All data were expressed as means ± SEM. To assess parallelism between androstenedione standards and serially diluted bull plasma samples containing endogenous androstenedione, a non-linear regression test was performed. Pearson’s correlation test was used to analyze the statistical relation between androstenedione concentrations estimated by EIA and RIA procedures. Significance was considered at P < 0.05 level if otherwise not stated.

## Results and discussion

### Assay validation

**Figure 1:**
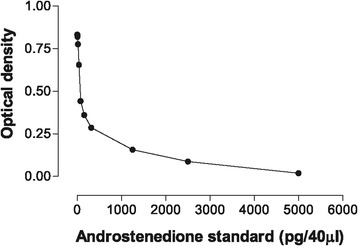
**Standard Curve for an enzyme immunoassay to determine plasma androstenedione in bulls.**

The lowest androstenedione detection limit significantly different from zero concentration was 0.2 pg/well/40 μl plasma, which corresponded to 5 pg/ml plasma (Figure 1). To determine the possible interference of plasma with the assay sensitivity, androstenedione standards in various amounts of charcoal treated plasma (10, 20 and 40 μl) were run in an assay. The EIA was carried out taking duplicates of 40 μl of unknown plasma samples and androstenedione standards ranging from 0.20 to 200 pg/40 μl/well. The sensitivity of EIA in the present experiment was 5 pg/ml of plasma, while previous studies employing RIA reported the sensitivity to be 20 pg/ml [10], 300 pg/ml [11] and 8 pg/ml [4]. Peripheral plasma androstenedione concentrations determined in bull calves (104–990 pg/ml) were similar as reported by others [12,13].

### Intra-and inter-assay precision

**Figure 2:**
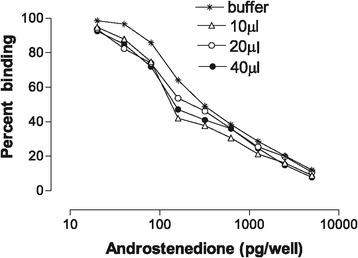
**Androstenedione standard curve (Percent binding) as influenced by buffer and different volumes of plasma.**

**Figure 3:**
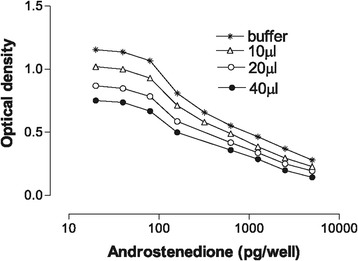
**Androstenedione standard curve (Optical density) as influenced by buffer and different volumes of plasma.**

The precision of the technique based on the intra- and inter-assay coefficients of variation (CV) was found to be 8 and 9%, respectively. Different plasma volumes for the EIA (viz. 10, 20 and 40 μl) did not influence the shape of the standard curve even though a slight drop in the OD_450_ was seen with higher plasma volumes (Figures 2 and 3).

### Specificity

The androstenedione antiserum (A1 pool 1, anti- Δ^4^-6-HS) used in these assays was highly specific. The cross-reactivity of the anti-Androstenedione serum against different related steroids was assessed to quantify the specificity of the androstenedione antisera (Table 1).

**Table 1:** **Cross-reactivity of androstenedione antibody**

**Steroids**	**Cross reaction** ^**a**^
Androstenedione	100
Androsterone	56
Δ-Androsten-3β-17β-diol	<0.001
1,4-androstadien-17β-ol-3-one	0.4
5-androsten-3β-ol-17-one (dehydroisoandrosterone)	22
5α-androstan-17β-ol-3-one (androstanolone)	1
Testosterone	1.3
17α-methyl testosterone	<0.001
Epitestosterone	0.1
Ethinylestradiol	<0.001
5α-dihydroandrostenedione	10
Estradiol 17-β	<0.001
Cortisol	<0.001
progesterone	0.4

^a^percentage cross-reaction relative to androstenedione at 50% binding.

### Recovery of the added hormone concentrations

Recoveries (mean ± SE) of known amounts androstenedione previously added to androstenedione free plasma (100, 200, 400, 1000 and 2000 pg/ml) were 102.24 ± 2.14, 198.45 ± 4.67, 396.76 ± 5.63, 1006.65 ± 8.27 and 2009.41 ± 11.16 pg/ml, respectively.

### Parallelism between androstenedione standards and endogenous androstenedione in bull plasma

**Figure 4:**
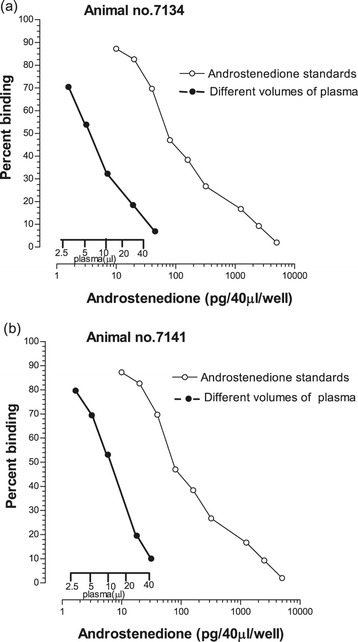
**Parallelism for bovine androstenedione standards with serially diluted plasma volumes of Animal no.7134 (a) and Animal no. 7141 (b).**

A higher degree of parallelism (*P* < 0.01) was obtained for androstenedione standard curve with serially diluted bull plasma samples containing high concentrations of endogenous androstenedione (Figure 4). A parallel drop was seen in percent binding with increasing plasma volume size and bovine androstenedione standards.

### Comparison with RIA

**Figure 5:**
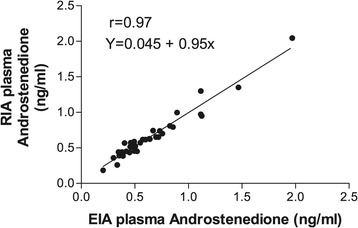
**Correlation between plasma androstenedione concentrations obtained by RIA and EIA.**

EIA procedure described above was compared with the values obtained employing the RIA procedure.The concentrations of the hormone obtained by the two methods were very similar and highly correlated (Figure 5).

## Conclusions

In conclusion, we described a specific, sensitive, androstenedione enzyme immunoassay standardized and validated for bovine plasma. The validated androstenedione EIA provides a reliable alternative to RIA. Apart from being non-radioactive in nature, the procedure is safe to perform, cost effective and less time-consuming than the conventional RIA procedures; it can also be adopted in laboratories where financial constraints limit the adoption of RIA.
